# Spz/Toll-6 signal guides organotropic metastasis in *Drosophila*

**DOI:** 10.1242/dmm.039727

**Published:** 2019-10-01

**Authors:** Ketu Mishra-Gorur, Daming Li, Xianjue Ma, Yanki Yarman, Lei Xue, Tian Xu

**Affiliations:** 1 Howard Hughes Medical Institute, Department of Genetics, Yale University School of Medicine, New Haven, CT 06519, USA; 2 School of Life Sciences, Westlake University, 18 Shilongshan Road, Hangzhou 310024, China; 3 Department of Neurosurgery, Yale University School of Medicine, New Haven, CT 06519, USA; 4 Shanghai Key Laboratory for Signaling and Diseases, School of Life Science and Technology, Tongji University, Shanghai 200092, China

**Keywords:** JNK, Tumor, Cancer, Toll-like receptors, Spätzle, Cell migration

## Abstract

Targeted cell migration plays important roles in developmental biology and disease processes, including in metastasis. *Drosophila* tumors exhibit traits characteristic of human cancers, providing a powerful model to study developmental and cancer biology. We now find that cells derived from *Drosophila* eye-disc tumors also display organ-specific metastasis, invading receptive organs but not wing disc. Toll receptors are known to affect innate immunity and the tumor inflammatory microenvironment by modulating the NF-κB pathway. Our RNA interference (RNAi) screen and genetic analyses show that Toll-6 is required for migration and invasion of the tumor cells. Further, receptive organs express Toll ligands [Spätzle (Spz) family molecules], and ectopic Spz expression renders the wing disc receptive to metastasis. Finally, Toll-6 promotes metastasis by activating JNK signaling, a key regulator of cell migration. Hence, we report Toll-6 and Spz as a new pair of guidance molecules mediating organ-specific metastatic behavior and highlight a novel signaling mechanism for Toll-family receptors.

## Abstract (precis)

**Summary:** The authors report Toll-6 and Spätzle as a new pair of guidance molecules mediating organ-specific metastasis behavior by activating JNK signaling, highlighting a novel role for Toll-family receptors.

## INTRODUCTION

Targeted cell migration and invasion play important roles in a variety of biological and disease processes. This phenomenon is exemplified by organotropic metastasis in cancer progression. Tumor metastasis is the leading cause of death for cancer patients (Lambert et al., 2017). A fundamental feature of metastasis is the ability of distinctive tumor types to colonize different organ sites, which depends on the inherent properties of the tumor cells and their interaction with the host tissue. However, the mechanism of organotropic metastasis is not well delineated. Deciphering the underlying molecular mechanism(s) of interactions between tumor cells and secondary host tissue is essential for our understanding of metastasis.

Almost a century ago, two theories were stated to explain organotropic tumor metastasis. James Ewing described the ‘anatomical mechanical theory’ to account for cancer metastasis (Ribatti et al., 2006). He theorized that the patterns of blood flow from the primary tumor can predict the first metastasized organs. On the other hand, Stephen Paget's ‘seed and soil’ theory hypothesized that tumor cells migrate to tissues that support their growth (Fidler, 2003). In other words, he suggested that the site of metastasis depended on the affinity of the tumor for the microenvironment. A modern version of the seed and soil theory focuses on a ‘homing mechanism’, which suggests that tumor cells are drawn to specific organ sites because of complex signaling crosstalk between the tumor cells and the cells of the organ (Fidler, 2002). Consistent with this idea, recent studies in mammalian systems indicate that chemokines and their receptors are involved in organ-specific tumor metastasis (Takeuchi et al., 2007; Zlotnik, 2006).

The strength of forward genetic screens and mosaic analysis in *Drosophila* has empowered investigators to utilize this model organism for identifying genes of cancer relevance, defining cancer signaling pathways and deciphering cancer biology. We have previously performed a genetic screen and developed a *Drosophila* model for tumor metastasis (Pagliarini and Xu, 2003). Somatic cells expressing oncogenic Ras protein (Ras^V12^) and simultaneously carrying a loss-of-function mutation in any of the cell polarity genes, including *scribbled* (*scrib*), *lethal giant larvae* (*lgl*) or *discs large* (*dlg*), develop into malignant tumors (known as *Ras^V12^*/cell-polarity-defect tumors). This fly tumor model displays the main hallmarks of human metastatic cancers, including uncontrolled growth, basement membrane (BM) degradation, loss of E-cadherin, migration, invasion, and secondary-tumor formation at distant organs. Further studies have revealed that JNK (c-Jun N-terminal kinase) signaling is activated in the tumor cells and is required for tumor-cell migration and invasion (Ma et al., 2013, 2014; Igaki et al., 2006). We also learned that JNK signaling can be activated non-autonomously or under stress conditions and can propagate to collaborate with *Ras^V12^*-expressing cells to induce tumorigenesis (Wu et al., 2010).

In this study, we have conducted a pathological and chronological examination of tumor progression and invasion in the fly tumor model. We have discovered that tumor cells metastasize to distal organs in a tissue-specific pattern. To identify genes responsible for organotropic metastasis, we successfully established fly tumor cell lines from the imaginal disc of a single larva and performed a genome-wide RNA interference (RNAi) screen. We identified Toll-6, a Toll-receptor family member, as a crucial gene for tumor cell migration. We also show that Toll-6 is required in tumor cells for organ-specific metastasis *in vivo* by inducing JNK signaling activation. Finally, we have discovered that the expression of the Spätzle (Spz) ligands in targeted organs serves as a cue for the guided migration and invasion. The Spz/Toll-6 system provides a novel molecular mechanism for organotropic metastasis.

## RESULTS

### Organ-specific metastasis of epithelial tumors in *Drosophila*

**Fig. 1.**
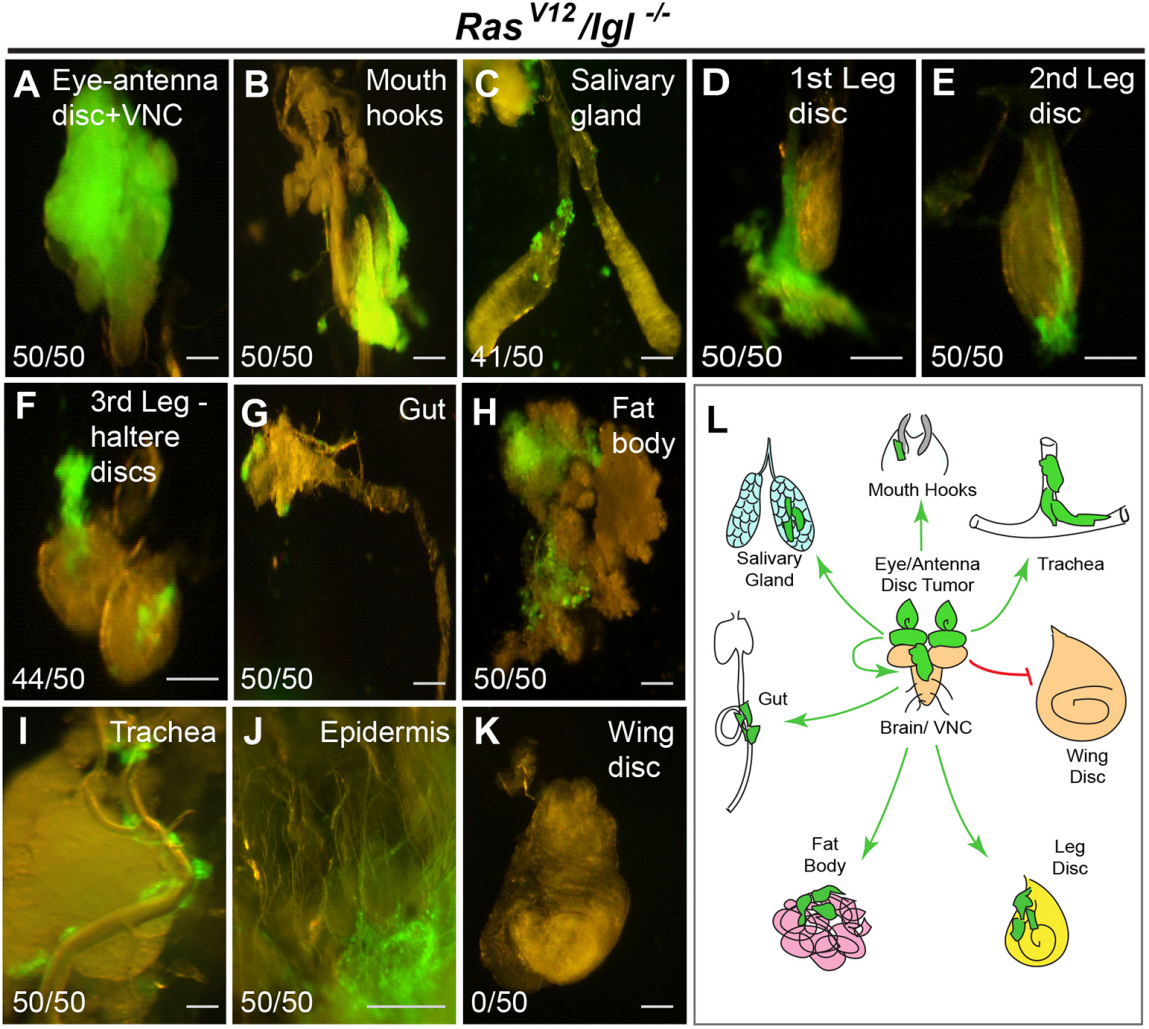
**Organotropic** **metastasis behavior in *Drosophila* neuro-epithelial tumors.** (A-K) GFP-labeled *Ras^V12^/lgl^−/−^* clones were generated in eye discs. The ensuing clones develop into tumors and metastasize onto the ventral nerve cord (VNC; A), mouth hooks (B), salivary glands (SGs; C), first leg disc (D), second leg disc (E), third leg disc and haltere disc (F), gut (G), fat body (FB; H), trachea (I) and skin (J). Note that tumor cells do not metastasize onto the wing disc (K). Numbers at bottom left of each panel indicate number of larvae displaying metastasis to a particular organ out of the total number of larvae examined. (L) A schematic representation of the organotropic metastasis pattern in *Drosophila*. Scale bars: 50 μm.

We have previously shown that, in *Drosophila* eye-antennal discs, oncogenic *Ras* (*Ras^V12^*) or *Raf* (*Raf^gof^*) can cooperate with mutants that disrupt cell polarity, including *scrib*, *dlg* or *lgl*, to induce tumor invasion and metastasis (Pagliarini and Xu, 2003). These fly tumors display uncontrolled proliferation, BM degradation, migration, invasion and secondary-tumor formation, which are characteristic of human tumors (Igaki et al., 2006). To further utilize *Drosophila* as a model system to explore the underlying *in vivo* mechanism of tumor metastasis, we performed a detailed study of the pathology of epithelial tumor dissemination and investigated the spatial and temporal pattern of tumor cell migration and invasion using macro- and microscopic methods. Previously, we have shown that these tumors metastasize at around day 7 after egg laying and progressively invade other tissues until larval death at approximately day 15 (Pagliarini and Xu, 2003). We discovered that the appearance of secondary tumors gradually radiates away from the primary tumors in the eye-antennal disc, suggesting a more directed migration rather than the hemolymph being the main route for metastasis. Hence, we examined early-stage larvae and progressively followed their metastasis pattern from day 7 to day 12. We found that tumor cells initially migrate anteriorly towards the mouth hooks and posteriorly towards the ventral nerve cord (VNC), both of which are physically attached to the eye-antennal disc, the host tissue of the primary tumors (Fig. 1A,B; Figs S1A,B,J and S2A,B). Strikingly, we also observed an organotropic tumor metastatic behavior. The tumor cells never invade or migrate onto the wing disc (Fig. 1K; Figs S1G and S2I) while readily metastasizing onto other tissues, including the VNC, mouth hooks, salivary glands (SGs), leg and haltere discs, gut, and fat body (FB) (Fig. 1A-J,L; Figs S1A-S‴ and S2C-H).

**Fig. 2.**
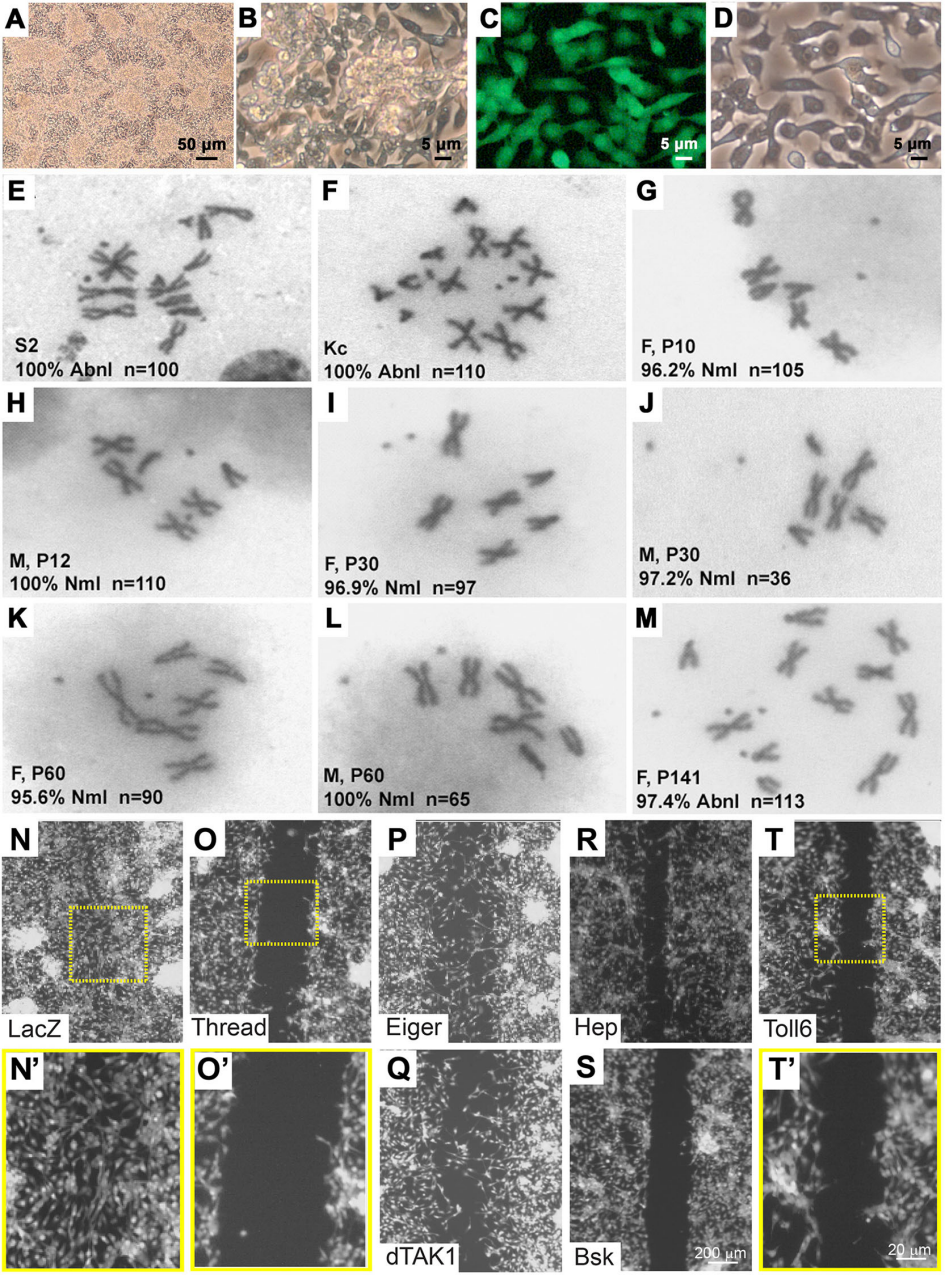
**Establishment and characterization of fly tumor cell lines and RNAi screen.** (A,B) *Ras^V12^/scrib^−/−^* tumor cell lines at low (A) and high (B) magnification. (C,D) Fluorescent (C) and bright-field (D) image of *Ras^V12^/scrib^−/−^* tumor cells. (E-M) Karyotyping of various cultured cell lines. All (100%) S2 (E) and Kc (F) cells exhibit abnormal chromosomes (Abnl). *Ras^V12^/scrib^−/−^* female cells and male cells possess a normal karyotype (Nml) at early passages (G-L) and abnormal karyotype after a long period in culture (P141: M, tetraploidy except for third chromosome). We noticed that the female-derived cells are more prone to losing one of the X chromosomes (data not shown). (P: passage number; n: number of cells examined; ‘F’: females; ‘M’: males). (N-T′) *Ras^V12^/scrib^−/−^* cells were used in an *in vitro* scratch assay to perform a genome-wide screen. Negative control: *lacZ* RNAi (N,N′); positive control: *Thread* (*Diap1*) RNAi (O,O′). Knockdown of Eiger (P), dTAK1 (Q) and Hep (R) partially blocked migration of cells, whereas knockdown of Bsk (S) and Toll-6 (T,T′) dramatically blocked tumor cell migration. Yellow boxes indicate area of higher magnification corresponding to dashed boxes.

To dissect the molecular events underlying this organotropic tumor metastatic behavior observed *in vivo*, we developed an *in vitro* culture system. We established cell lines of various genotypes from *Drosophila* epithelial tumors derived from either the eye- or wing-disc and optimized their culture conditions (Fig. 2A-D, Fig. S3A-F). The tumor cell lines were derived from single larvae, thus minimizing genotypic variability (see Materials and Methods). Karyotype analysis revealed that, in contrast to Schneider 2 (S2) and Kc cells, which showed 100% penetrance of chromosomal aneuploidy (Fig. 2E,F), the karyotype of tumor cell lines remains largely normal at passages as late as P60 (Fig. 2G-L, Fig. S4), but extensive culture results in partial chromosomal abnormalities (Fig. 2M, Fig. S4). These tumor cell cultures with a uniform genetic background were then used to establish clonal *Ras^V12^/scrib^−/−^* mutant cell lines by fluorescence-activated cell sorting (FACS) and dilution methods (see Materials and Methods). Twelve percent of the single cells from FACS sorting (48/400) were successfully cloned (Fig. S3G,H) and tested for ease of application to various genetic and biological manipulations. Additionally, the cells are easily transfectable and phenotypically display invasive behavior both *in vitro* and *in vivo* (Figs S5, S6, S7), highlighting the versatile potential of these fly tumor cell lines.

### Spz/Toll-6 axis regulates organotropic metastatic behavior

**Fig. 3.**
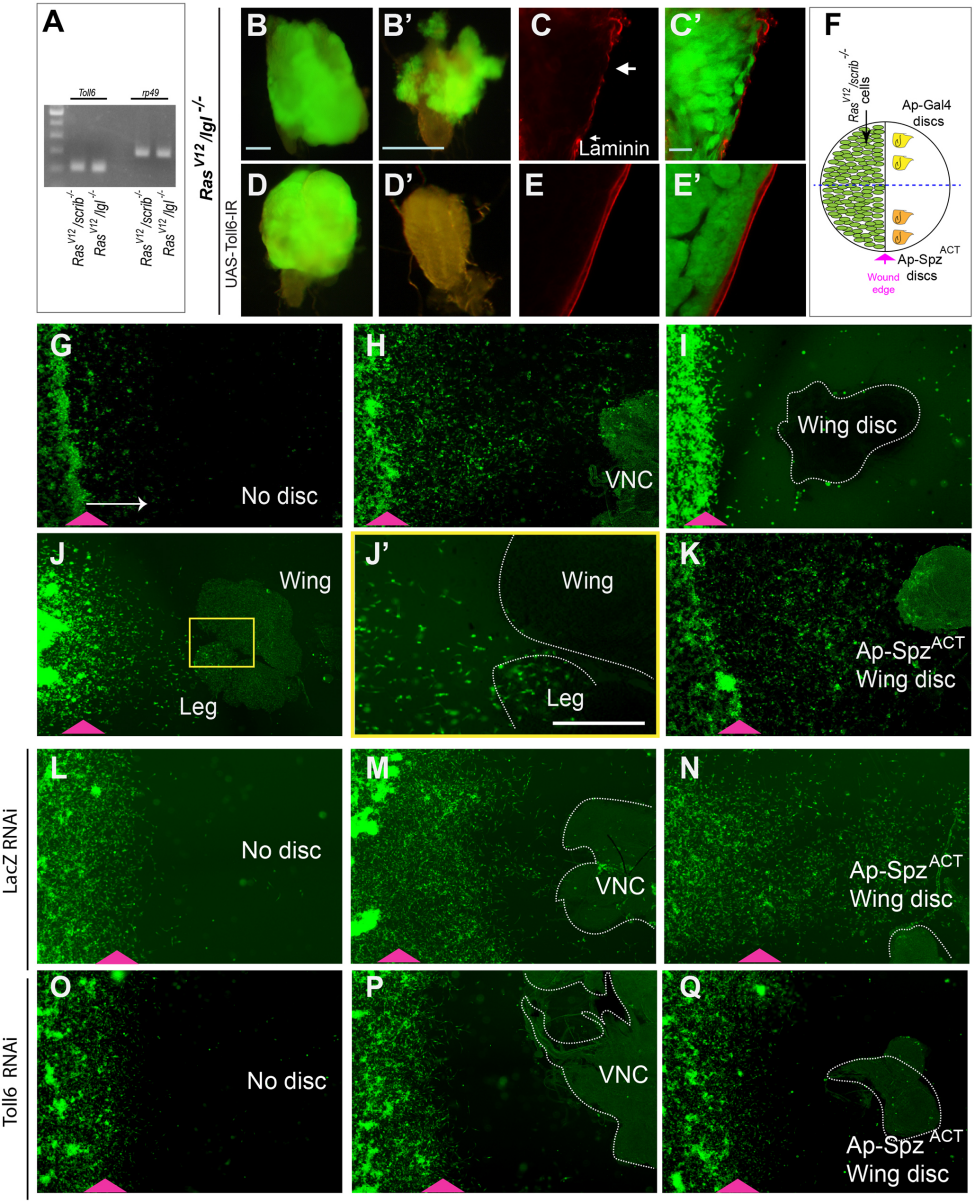
**Toll-6 mediates migration of tumor cells *in vivo* and *in vitro*.** (A) RT-qPCR to determine expression of Toll-6 in *Ras^V12^/scrib^−/−^*- and *Ras^V12^/lgl^−/−^*-induced tumors. RP49 is used as an internal control. (B-E) *Ras^V12^/lgl^−/−^*-induced tumor invasion (B,B′) and BM degradation (C,C′) are blocked by *Toll-6* RNAi (D-E′); C,C′,E,E′ show margin of tumor stained for LaminA/C (red). Arrows indicate BM degradation. (F) Schematic representation of a test plate for *in vitro* migration assay: a confluent lawn of *Ras^V12^/scrib^−/−^* cells in a 35 mm tissue-culture dish is scratched, two wild-type (WT) wing discs or wing discs expressing Spz^ACT^ are placed in the same dish. (G-K) Within 24 h, tumor cells migrate towards the VNC (H) or wing disc expressing Spz^ACT^ (K), but not WT wing disc (I). All tissues (controls and test) were placed in the same dish to minimize variability and each experiment repeated at least three times; a representative experiment is shown. When leg disc was placed adjacent to a wing disc, tumor cells selectively migrate to the leg disc (J,J′). Yellow panel: higher magnification of boxed area. (L-Q) Knockdown of Toll-6 but not *lacZ* in *Ras^V12^/lgl^−/−^* tumor cells blocked *in vitro* organotropic metastasis towards VNC (compare M and P) or Spz^ACT^-expressed wing discs (compare N and Q). (G-Q) Arrowhead (pink) indicates edge of wound/scratch. Arrow in G indicates the direction of migration. Cells that migrated beyond 200 µm (indicated by the length of the arrow) were counted and graphed (see Fig. S8). Scale bars: B,B′,D,D′: 200 µm; C,C′,E,E′: 50 µm; J′: 200 µm; G-Q: arrow equals 200 µm.

We then performed a genome-wide RNAi screen using these *Ras^V12^/scrib^−/−^* tumor cells to identify the potential receptor responsible for organ-specific tumor cell migration (Echeverri and Perrimon, 2006). In the scratch assay (Hall, 2005), the fly tumor cells were found to be highly migratory, capable of covering the entire scratch area within 22 h (Fig. 2N). Multiple core components of the JNK pathway were identified in this screen, including *eiger* (*egr*), *dTAK1*, *hemipterous* (*hep*) and *basket* (*bsk*) (Fig. 2P-S), in accordance with their roles in promoting tumor metastasis (Igaki et al., 2006). One of the remaining candidates is Toll-6, a member of the Toll-family receptors (Tauszig et al., 2000), as knockdown of Toll-6 dramatically blocked tumor cell migration (Fig. 2T,T′). Toll-6 expression is detected at both the RNA and protein level in *Ras^V12^/scrib^−/−^* as well as *Ras^V12^/lgl^−/−^* tumors (Fig. 3A, Fig. S8A-E′). Consistent with our *in vitro* data, while having a minor effect on tumor growth, expression of the *Toll-6* dsRNA (RNAi) in the tumor-bearing flies dramatically blocks metastasis and tumor-induced BM degradation (Fig. 3B-E′). Furthermore, like the *Ras^V12^/scrib^−/−^* tumors, the *Ras^V12^/lgl^−/−^* tumors also grow and encounter other organs. However, unlike the invasive tumors, *Toll-6-IR* (*Toll-6* RNAi) tumors do not invade these organs, which can be easily and cleanly separated away from the tumor tissue (Fig. S8F-F″).

Spz serves as the ligand for Toll receptors and activates downstream signaling events (Lewis et al., 2013; Parthier et al., 2014; Gangloff et al., 2008). Since Toll-6 is required in the tumor cells for metastasis, we asked whether Spz and related molecules are expressed on the target organs and serve as an attractant for the tumor cells. Several larval tissues are known to express *spz* mRNA, including the FB, SG and hemocytes (Irving et al., 2005; Meyer et al., 2014; Shia et al., 2009). In addition to Spz, there are five Spz-related genes in the *Drosophila* genome (Parker et al., 2001) that could encode potential ligands for the Toll-family receptors. We performed reverse-transcription PCR (RT-PCR) and found that several Spz-related genes are expressed in metastasis-receptive organs, including VNC, mouth hook, SG, leg disc, FB and gut, but not in the non-receptive wing discs (Fig. S9A).

Next, to test whether Spz could serve as an attractant for Toll-6-mediated metastasis, we developed an *in vitro* culture system, similar to the one used for studying neuronal migration (Jia et al., 2005). Briefly, freshly dissected individual organs were placed adjacent to tumor cell lines in culture medium, and the migration process of tumor cells towards the specific organ was examined (Fig. 3F). Using this assay, we can recapitulate the organ-specific metastasis pattern observed *in vivo*. Within 24 h, we can clearly detect migration of the tumor cells towards the VNC (Fig. 3H), which is distinctly different from the random migration of the tumor cells in culture (Fig. 3G). Similar migration and invasion patterns are observed for all the organs that are invaded by tumor cells *in vivo*, including leg disc, haltere disc, FB, trachea and SG (Fig. 3J,J′ and data not shown). In sharp contrast to the migration of cells towards receptive organs like the VNC, when the wing disc was examined in this assay we found no directed migration of tumor cells towards the disc (Fig. 3I). In fact, when both leg and wing discs are placed together in the same assay, tumor cells preferentially migrate towards the leg disc (Fig. 3J,J′). This suggests that the dissemination of the *Ras^V12^*/cell-polarity-defect tumors in *Drosophila* is an active migratory process, which exhibits organ-specific metastatic behavior (Fig. 1L). To further investigate whether Spz serves as a guide for Toll-6-mediated organotropic metastasis, we overexpressed an activated form of Spz (Spz^ACT^) in the wing disc under the *apterous* (*ap*) promoter (Ligoxygakis et al., 2002) and used it in our *in vitro* culture system. In contrast to wild-type wing disc, tumor cells migrated towards the Spz^ACT^-expressing wing disc and invaded it within 24 h (Fig. 3K). When the Spz^ACT^-expressing wing disc was placed on a lawn of semi-confluent tumor cells, the cells immediately adjacent to the receptive tissue never displayed random migration. Instead, they migrated onto the disc and accumulated on the tissue (Fig. S9B′, Fig. S10). Interestingly, in the case of the control wing disc, the cells migrated onto the attached tissue (nerves, tracheal fibers) but not onto the disc surface (Fig. S9B). This observation supports the role of Spz as a chemoattractant. To further test whether Toll-6 was serving as the corresponding receptor in the tumor cells responding to Spz^ACT^, we used RNAi to downregulate *Toll-6* expression in the cells and assessed their migration towards Spz^ACT^-expressing wing discs. Compared with cells treated with *lacZ* dsRNA for RNAi (Fig. 3L-N), tumor cells treated with *Toll-6* dsRNA displayed a dramatically reduced migration towards both the VNC as well as Spz^ACT^-expressing wing disc (Fig. 3O-Q; for quantification of cell migration see Fig. S9C,D). Together, these data indicate that the Spz/Toll-6 axis serves as a molecular apparatus mediating organ-specific metastasis in *Drosophila* and strongly suggest the existence of underlying molecular machinery guiding the targeted migration and invasion of tumor cells.

### Toll-6 activates JNK signaling

**Fig. 4.**
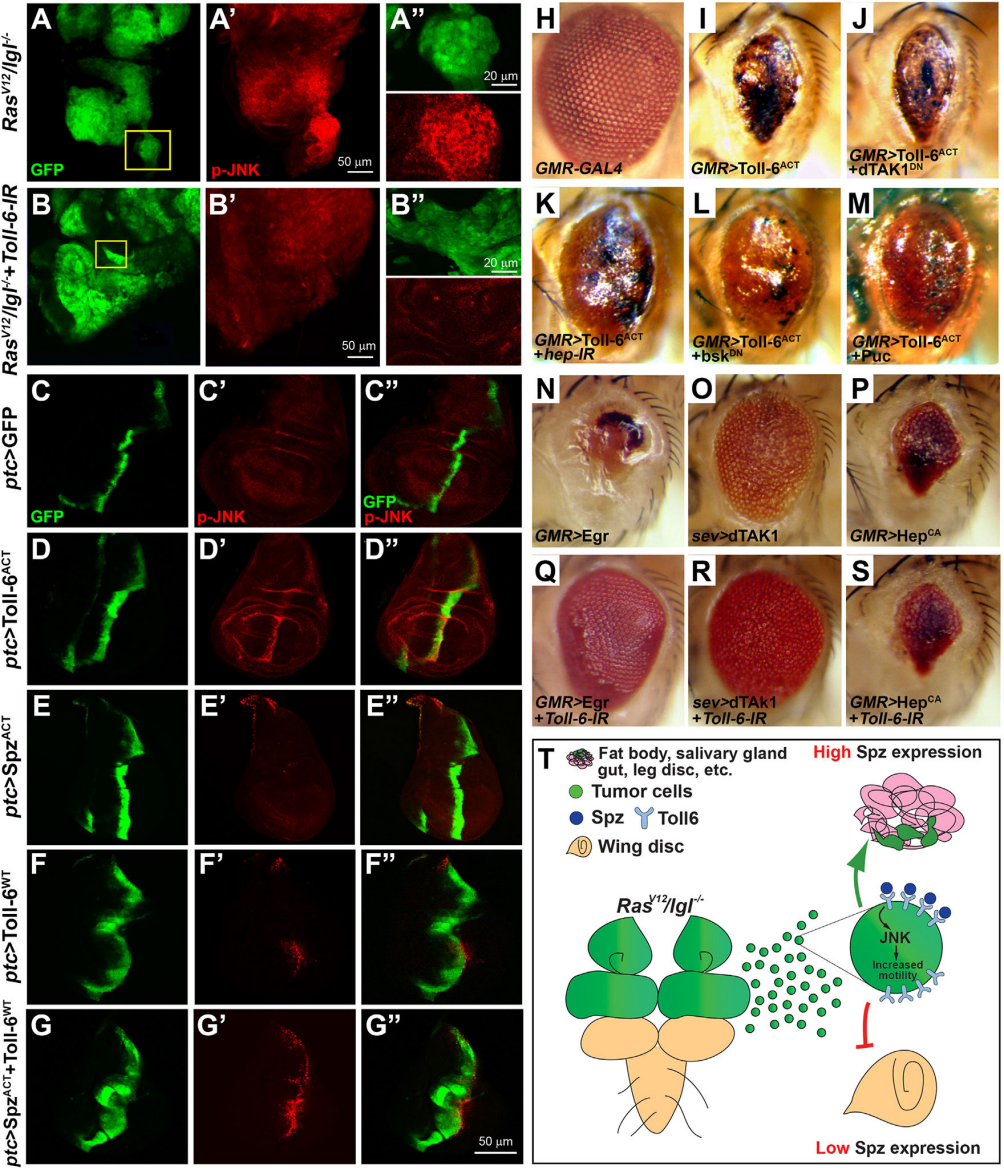
**Toll-6 activates JNK signaling.** (A-B′′) Knockdown of Toll-6 reduces *Ras^V12^/lgl^−/−^* tumor-induced JNK activation, as shown by phosphorylated JNK (p-JNK) staining. (C-G″) p-JNK staining of wing discs under *patched* promoter. Compared with control (C-C″), ectopic expression of activated Toll-6 (Toll-6^ACT^) activates JNK (D-D″). (E-G) Co-expression of Spz^ACT^ strongly increased wild-type Toll-6-induced JNK activation (F-G″), whereas expression of Spz^ACT^ alone is not sufficient to activate JNK (E-E″). (H-S) Light micrographs of *Drosophila* adult eyes are shown. Compared with the control (H), ectopic expression of activated Toll-6 under the *GMR* promoter induces a small-eye phenotype (I), which is suppressed by expression of a *Hep* dsRNA (K), a dominant-negative form of Bsk (L), or Puc (M), but not by that of a dominant-negative form of dTAK1 (J). Knockdown of Toll-6 suppresses ectopic Egr- or dTAK1-expression-induced small-eye phenotypes (N,O,Q,R) but not that of Hep^CA^ expression (P,S). (T) A working model of Spz/Toll-6 in guiding organotropic metastasis in *Drosophila*.

Given that JNK signaling is required for cell migration and its inhibition blocks metastasis (Igaki et al., 2006, 2009), we reasoned that Toll-6 could mediate metastasis by regulating JNK activation. Indeed, knockdown of Toll-6 significantly reduces JNK activation as revealed by phospho-JNK (p-JNK) immunostaining and western blotting (Fig. 4A,B; Fig. S11A). In addition, ectopic expression of activated Toll-6 (Toll-6^ACT^) in the wing disc results in JNK activation (Fig. 4C-D″). Hence, to test whether Spz could trigger Toll-6-mediated JNK activation, we ectopically expressed Spz^ACT^ and wild-type Toll-6 (Toll-6^WT^) in wing discs under *ptc*-*Gal4*. Although expression of Spz^ACT^ or Toll-6^WT^ alone induces no or mild JNK activation, respectively (Fig. 4E-F″), co-expression of both strongly induces JNK activation (Fig. 4G-G″), suggesting that Spz can serve as a ligand for Toll-6 to activate JNK signaling. Furthermore, in accordance with the observation that JNK activation is associated with endocytic vesicles (Igaki et al., 2009), double staining reveals that Toll-6^ACT^ colocalizes with p-JNK (Fig. S11B-D). Interestingly, a closer examination also revealed that co-expression of Spz^ACT^ and Toll-6^WT^ synergistically induces cell invasion behavior, as GFP-positive cells collectively migrate away from the anterior-posterior boundary and towards the posterior part of the wing disc (Fig. S12A-C″), phenocopying JNK activation, as has been reported previously (Ma et al., 2014, 2017). Finally, it has been reported that, although there is a certain redundancy in the ligands that activate Toll-6, Spz5 is the main ligand for Toll-6 (McIlroy et al., 2013). Since we detected that Spz^ACT^ can activate JNK via Toll-6, we then also tested the ability of Spz5 to activate Toll-6, and hence JNK, *in vivo*. We find that, although Spz5 overexpression is sufficient to induce a slight JNK activation (Fig. S12D″) (Foldi et al., 2017), it can significantly increase wild-type Toll-6-mediated JNK activation and cell migration (Fig. S12A-E″). Taken together, these data demonstrate that Toll-6 activates JNK signaling *in vivo*.

Next, to further delineate the action of Toll-6 in the JNK pathway, we performed genetic analysis between Toll-6 and the known components of the JNK signaling pathway. We overexpressed activated Toll-6 in the developing eye using the *GMR*-*Gal4* driver (*GMR*>*Toll-6^ACT^*), which produces a small-eye phenotype (Fig. 4H,I). This resembles the effect of expressing a constitutively active form of Hep (Hep^CA^), a JNK kinase (Fig. 4P), consistent with the notion that Toll-6 activates JNK signaling. The Toll-6^ACT^ eye phenotype is suppressed by knocking down Hep (Fig. 4K), by expression of a dominant-negative form of *bsk* (*Drosophila* JNK; Fig. 4L) or by expression of JNK inhibitor Puckered (*Puc*; Fig. 4M). However, it is not affected by inactivation of an upstream component of the pathway, dTAK1 (Fig. 4J). Consistently, knockdown of *Toll-6* suppresses the small-eye phenotype induced by ectopic expression of Egr or dTAK1, but not that of Hep^CA^ (Fig. 4N-S). Collectively, these genetic data suggest that Toll-6 acts in the JNK signaling pathway upstream of Hep and Bsk.

## DISCUSSION

### A *Drosophila* model for organotropic metastasis

Metastasis is the leading cause of mortality in cancer patients. Befittingly, both the words ‘cancer’ (Latin: ‘crab’) and ‘metastasis’ (Greek: ‘displacement’) refer to cell movement: the crab-like invasion of cancer into healthy tissue and the migration of cancer cells to secondary sites (Murphy, 2001). Since Paget's initial observation more than 100 years ago, pathologists have recognized that the movement of cancer cells is not random and that different types of cancer have different destinations or organ-specific metastasis (Murphy, 2001). For example, colon carcinomas usually metastasize to liver and lung but rarely to bone, skin or brain and almost never to kidneys, intestine or muscle (Obenauf and Massagué, 2015). In contrast, other tumors, such as breast carcinomas, frequently form metastases in most of these organs, while prostate cancer metastasis occurs most prominently in bone (Ribatti et al., 2006). Here, we report a similar phenomenon in the spread of malignant tumors in *Drosophila*. The *Ras^V12^*/cell-polarity-defect tumors exhibit organ-specific metastasis. Tumor cells originating from the eye-antennal imaginal disc in the larvae metastasize to almost all organs (mouth hook, VNC, SG, leg discs, haltere disc, gut, FB) except the wing disc.

In mouse cancer models and human patients, it is known that tumor cells are disseminated through vascular networks such as the blood and lymphatic vessels (Karaman and Detmar, 2014). The tracheal system in *Drosophila* is a tubular network for supplying oxygen, functioning as the equivalent of human lungs and blood vessels. Interestingly, in flies, we also observed that tumor cells from primary tumors can metastasize to the trachea, suggesting that the trachea might function as an essential media to facilitate tumor metastasis. Consistent with this hypothesis, a recent study by Ross Cagan and Benjamin Levine showed that trachea-derived tumor cells can migrate significant distances (Levine and Cagan, 2016). Additionally, *Ras^V12^*/cell-polarity-defect tumors have been shown to express tracheal markers and migrate along the trachea (Grifoni et al., 2015). It will be interesting to further study the role of the trachea in organotropic metastasis, and possibly investigate whether a similar conserved mechanism exists in mammalian tumor cell migration.

### Spz/Toll-6: a new nexus for guiding targeted migration of tumor cells

Targeted cell migration plays a key role in normal development. Studies of neural development have identified multiple receptors and their ligands that regulate guided neuronal migration (e.g. Robo/Slit, Trk receptors/neurotrophins) (Andrews et al., 2007; Puehringer et al., 2013). For organotropic metastasis, Paget proposed the ‘seed and soil’ theory, which states that metastases develop only when the ‘seed’ (tumor cells) and the ‘soil’ (target organs) are compatible (Paget, 1989; Fidler et al., 2002). It is only recently that studies have begun to reveal some of the identities of the ‘seed and soil’ molecules that regulate the spread of tumor cells. Experiments with breast cancer cell lines showed that inhibition of the chemokine receptor CXCR4 by a neutralizing antibody abrogates their metastasis to the lung, which expresses the corresponding chemokine ligand, CXCL12 (Muller et al., 2001). However, the CXCR4/CXCL12 interaction is unlikely involved in the metastasis of breast cancers to the liver (Wang et al., 2006). The diverse types of cancers and their complex metastatic patterns indicate that other ‘seed and soil’ molecules must be involved in organ-specific metastasis.

Here, we report that Toll-6, a Toll-family neurotrophin receptor, plays an essential role in tumor cell metastasis in *Drosophila*. Our study shows that Toll-6 is expressed in the *Ras^V12^/*cell-polarity-defect tumors and that downregulation of Toll-6 in the tumor cells blocks their migration and invasion. Interestingly, we also showed that Toll-6-expressing tumor cells migrate towards and invade the organs that express Spz or Spz-related molecules. Furthermore, the Toll-6-expressing tumor cells do not metastasize to the wing disc, an organ with no detectable Spz or Spz-related gene expression in our system, strongly arguing that Spz or a Spz-related molecule could be serving as a cue for guiding Toll-6-expressing tumor cells. Interestingly, a recent study by Alpar et al. also shows that the wing disc produces extremely low levels of Spz in a tightly regulated spatially restricted pattern with no resultant signaling events (Alpar et al., 2018). Indeed, artificial overexpression of Spz^ACT^ in the wing disc converts it to a tissue receptive for the migration and invasion by Toll-6-expressing tumor cells (Fig. S9B′). Together, these data indicate that Spz/Toll-6 serve as the ‘seed and soil’ molecules for organotropic metastasis in the fly. In support of our conclusion, clinical studies have revealed a correlation between increased expression levels of Toll-like receptors (TLRs) and malignancy of multiple cancers (O'Neill, 2008; Zhang et al., 2009). TLRs have also been shown to affect malignancy by altering the tumor inflammatory microenvironment (Fidler et al., 2007; Kim and Karin, 2011; Kim et al., 2009; Rakoff-Nahoum and Medzhitov, 2009). Given that the Toll-family receptors are evolutionarily conserved, consisting of nine members in *Drosophila* and ten TLRs in mammals (Yagi et al., 2010; Imler and Zheng, 2004), our study raises the possibility that mammalian TLRs could play a similar role in mediating organotropic metastasis and cell migration.

In *Drosophila*, Toll regulates dorsoventral patterning in embryos and anti-fungal defense in adults (Anderson et al., 1985; Lemaitre et al., 1996). Toll has also been found to have an inhibitory role in the formation of neuromuscular junctions (Halfon et al., 1995; Rose et al., 1997) and, recently, 18 wheeler, a Toll-like receptor protein, was found to play a role in border cell migration (Kleve et al., 2006). Studies in both flies and mammals show that Toll-family receptors mediate NF-κB signaling activation (Rakoff-Nahoum and Medzhitov, 2009; Brikos and O'Neill, 2008; Valanne et al., 2011). A study using biochemical inhibitors suggests that Toll molecules could affect random migration of neutrophils by activating ERKs (Aomatsu et al., 2008). Our study shows that Toll-6 activates JNK signaling, which is in accordance with the recent study by Foldi et al. that shows the role of Toll-6 and JNK in cell death (Foldi et al., 2017). However, JNK signaling is also a key regulator for cell migration during development (Davis, 2000), and we have previously shown that JNK signaling is activated in *Ras^V12^*/cell-polarity-defect tumors and is essential for metastasis (Igaki et al., 2006). Here, we report that, first, Toll-6 knockdown in *Ras^V12^*/cell-polarity-defect tumors completely blocks metastasis by effectively reducing JNK signaling; second, the role of JNK activation by Toll-6 is highlighted in the *in vitro* assay, as Toll-6 knockdown reduced migration of the tumor cells even when discs were not placed (Fig. 3, compare panels K and N; Fig. S9D), suggesting that JNK activation by Toll-6 might be important for general cell migratory behavior. Finally, ectopic expression of Toll-6^ACT^ in the wing disc results in JNK activation (Fig. 4D-D″). These data indicate that Toll-6 regulates metastasis by activating JNK signaling. Recently, Grindelwald (Grnd) has been identified as a novel tumor necrosis factor receptor (TNFR) mediating *Ras^V12^/scrib^−/−^*-induced tumor growth and invasion (Andersen et al., 2015). Our data here suggest that Toll-6 could be providing a second signal for the activation of JNK. Indeed, our epistasis data support the model that Toll-6 genetically interacts with the JNK pathway (Fig. 4H-S). It is possible that inputs from both Toll-6 and Grnd signals could result in or are required for a high level of JNK activation.

In addition to Spz, there are five Spz-related genes in the *Drosophila* genome, which could serve as ligands for Toll-6. Toll-6 has been recently reported to function as a neurotrophin receptor in regulating motor neuron targeting and survival, and it also physically binds to Spz5 (McIlroy et al., 2013; Foldi et al., 2017). Consistent with this, we found that co-expression of Toll-6 and Spz5 synergistically promote collective cell invasion in the developing wing, phenocopying Toll-6 and Spz^ACT^ co-expression (Fig. S12). Interestingly, although both Spz^ACT^ and Spz5 can activate Toll-6-mediated JNK signaling, we found that, unlike Spz5, expression of Spz^ACT^ alone is not sufficient to activate JNK (Fig. S12A-A″, D-D″). Given our results, we infer that Spz and Spz5 might display some redundancy. Indeed, a previous study by Foldi et al. has demonstrated the redundancy of Spz2 (DNT1) and Spz5 (DNT2) in binding to Toll-6 (Foldi et al., 2017). Furthermore, a redundancy between Spz, Spz2 and Spz5 has also been demonstrated by Sutcliffe et al. (2013), suggesting that Spz proteins may function as promiscuous ligands in some circumstances (e.g. upon overexpression) and can bind multiple Toll receptors. We believe that, under overexpression conditions, Spz is capable of activating Toll-6 (albeit at lower levels than is Spz5, as shown in Fig. S12). Therefore, as has been reported before (Sutcliffe et al., 2013), we also find that different Spz proteins might act redundantly to induce Toll-6-mediated JNK activation and cell migration. In conclusion, our genetic and biochemical data show that Toll-6 and Spz compose a new pair of guidance molecules for directing cell migration and that their interaction mediates organotropic metastasis by activating JNK signaling.

## MATERIALS AND METHODS

### Fly strains and generation of clones

Fluorescently labeled clones were produced in larval imaginal discs using the following strains: *y, w, eyFLP1; Act>y+>Gal4*, *UAS–GFP; FRT82B, Tub-Gal80* (82B tester) and *y, w, eyFLP1; Tub-Gal80, FRT40A; Act>y+>GAL4, UAS-GFP* (40A tester). Additional strains used were as follows: *GMR*-*GAL4*, *ptc-GAL4*, *ap-GAL4*, *sev-GAL4*, *UAS-GFP* and *puc^E69^* (*puc-lacZ*) were obtained from Bloomington *Drosophila* Stock Center. *UAS-spz^ACT^* was gift from J.-M. Reichart (Ligoxygakis et al., 2002). Five independent *UAS-Toll-6-IR* transgenic lines generated from three different constructs were obtained from Vienna *Drosophila* Resource Center (VDRC). *UAS*-*spz5^HA^* was obtained from FlyORF. *UAS-egr* (Igaki et al., 2002), *UAS-Hep^CA^*, *UAS*-*dTAK1*, *UAS-hep-IR*, *UAS-bsk^DN^*, *UAS*-*dTAK1*^*DN*^ and *UAS*-*puc* (Ma et al., 2012) were previously described. *UAS-Toll^WT^* and *UAS-Toll-6^ACT^-Flag* transgenic flies were generated by standard *P*-element-mediated transformation (Bestgene, Inc.). More than five independent lines were produced and examined for each transgene. Two RNAi lines (v27102 and v27103) were recombined and used to perform experiments unless indicated. Gene expression was verified by immunostaining.

### Genotypes for fly-related figures

The genotypes of flies for results shown in the figures are as shown below.

Fig. 1: (A-K) *y, w, ey-Flp/+; tub-Gal80, FRT40A/lgl^4^, FRT40A, UAS-Ras^V12^; Act>y+>Gal4, UAS-GFP/+*.

Fig. 3: (B,C) *y, w, ey-Flp/+; tub-Gal80, FRT40A/lgl^4^, FRT40A, UAS-Ras^V12^; Act>y+>Gal4, UAS-GFP/+*; (D,E) *y, w, ey-Flp/+; tub-Gal80, FRT40A/lgl^4^, FRT40A, UAS-Ras^V12^; Act>y+>Gal4, UAS-GFP/UAS-Toll-6-IR^V27102, V27103^*.

Fig. 4: (A-A″) *y, w, ey-Flp/+; tub-Gal80, FRT40A/lgl^4^, FRT40A, UAS-Ras^V12^; Act>y+>Gal4, UAS-GFP/+*; (B-B″) *y, w, ey-Flp/+; tub-Gal80, FRT40A/lgl^4^, FRT40A, UAS-Ras^V12^; Act>y+>Gal4, UAS-GFP/UAS-Toll-6-IR^V27102, V27103^*; (C) *ptc-Gal4, UAS-GFP/+*; (D) *ptc-Gal4, UAS-GFP/+; UAS-Toll-6^Act^/+*; (E) *ptc-Gal4, UAS-GFP/+; UAS-spz^Act^/+*; (F) *ptc-Gal4, UAS-GFP/UAS-Toll-6^WT^*; (G) *ptc-Gal4, UAS-GFP/UAS-spz^WT^; UAS-Toll-6^Act^/+*; (H) *GMR-Gal4/+*; (I) *GMR-Gal4, UAS-Toll-6^Act^/+*; (J) *GMR-Gal4, UAS-Toll-6^Act^/UAS-dTAK1^DN^*; (K) *GMR-Gal4, UAS-Toll-6^Act^/UAS-hep-IR*; (L) *GMR-Gal4, UAS-Toll-6^Act^/UAS-bsk^DN^*; (M) *GMR-Gal4, UAS-Toll-6^Act^/UAS-Puc*; (N) *UAS-Egr/+; GMR-Gal4/+*; (O) *sev-Gal4, UAS-dTAK1*/+; (P) *GMR-Gal4, UAS-Hep^CA^/+*; (Q) *UAS-Egr/+; GMR-Gal4/UAS-Toll-6-IR^V27102, V27103^*; (R) *sev-Gal4, UAS-dTAK1/+; UAS-Toll-6-IR^V27102, V27103^/+*; (S) *GMR-Gal4, UAS-Hep^CA^/UAS-Toll-6-IR^V27102, V27103^*.

Fig. S1: (A-I) *y, w, ey-Flp1; Act>y+>Gal4, UAS-GFP/+; FRT82B, tub-Gal80/UAS-Raf^GOF^, FRT82B, scrib^1^*; (J-O) *FRT19A, dlg^m52^/tub-Gal80, FRT19A; ey-Flp5, act>y+>Gal4, UAS-GFP/UAS-Ras^V12^*; (P-S‴) *y, w, ey-Flp/+; tub-Gal80, FRT40A/lgl^4^, FRT40A, UAS-Ras^V12^; Act>y+>Gal4, UAS-GFP/+*.

Fig. S2: *y, w, ey-Flp1; Act>y+>Gal4, UAS-GFP/UAS-Ras^V12^; FRT82B, tub-Gal80/FRT82B, scrib^1^*.

Fig. S3: (E) *y, w, hs-Flp1; Act>y+>Gal4, UAS-GFP/UAS-Ras^V12^; FRT82B, tub-Gal80/FRT82B, scrib^1^*.

Fig. S8: (A-A″) *ptc-Gal4, UAS-GFP/+*; (B-B″) *ptc-Gal4, UAS-GFP/UAS-spz^WT^*; (C-C″) *ptc-Gal4, UAS-GFP/UAS-spz^WT^; UAS-Toll-6-IR^V27102, V27103^*; (D-E′) *y, w, ey-Flp/+; tub-Gal80, FRT40A/lgl^4^, FRT40A, UAS-Ras^V12^; Act>y+>Gal4, UAS-GFP/+*; (F) *y, w, ey-Flp/+; tub-Gal80, FRT40A/lgl^4^, FRT40A, UAS-Ras^V12^; Act>y+>Gal4, UAS-GFP/UAS-Toll-6-IR^V27102, V27103^*.

Fig. S11: (B) *ptc-Gal4, UAS-GFP/UAS-Toll-6^WT^*; (C,D) *ptc-Gal4, UAS-GFP/+; UAS-Toll-6^Act^/+*.

Fig. S12: (A-A″) *ptc-Gal4, UAS-GFP/+; UAS-spz^Act^/puc^E69^*; (B-B″) *ptc-Gal4, UAS-GFP/UAS-Toll-6^WT^; puc^E69^/+*; (C-C″) *ptc-Gal4, UAS-GFP/ UAS-Toll-6^WT^; UAS-spz^Act^/puc^E69^*; (D-D″) *ptc-Gal4, UAS-GFP/+; UAS-spz5/puc^E69^*; (E-E″) *ptc-Gal4, UAS-GFP/UAS-Toll-6^WT^; UAS-spz5/puc^E69^*.

### Establishing *Drosophila* tumor cell lines

A single or 30 third-instar larvae were washed five times in a 10 cm Petri dish with sterile 1× PBS. The larvae were transferred into an Eppendorf tube, treated with four washes of 70% ethanol for 2 min followed by a brief vortexing, followed by rinsing twice with sterile 1× PBS. These wash steps were critical for preventing contamination in primary cultures. Using pre-sterilized forceps, the larvae were dissected in basic M3 FBS-free medium (Sigma-Aldrich) supplemented with 100 U/ml of penicillin, 100 µg/ml of streptomycin and 400 µg/ml of G418 (Life Technologies). The eye or wing discs with tumors were cut with a dissecting needle into several pieces and kept in an Eppendorf tube containing 1 ml of basic M3 medium with 400 µg/ml of G418 (100 µl medium for single-animal experiments). The tissues were washed once with 1× PBS, changed to 1 ml 1× trypsin-EDTA, and incubated at 37°C for 10 min (250 µl for single-animal experiments). After incubation, cells were drawn up and down by pipetting to dissociate cell clumps. Cells were spun at 1000 rpm (900 ***g***) for 5 min. The supernatant was removed, and the cells were resuspended in 1 ml cM3/FE medium (cM3 plus 2.5% fly extract) with 400 µg/ml of G418, before transfer to a 12.5 cm^2^ T-flask with 2 ml of medium (for single-animal experiments, 100 µl medium and a 96-well plate, respectively; for experiments using cM3/Ins or cM3/FE/Ins media, 0.125 IU/ml insulin was also added). Following overnight incubation of the cells at 25°C, medium was replaced with fresh medium at the same volume. Cell lines were then passaged at the ratio of 1:2 to 1:3 at the early passage (passages 1-3) every 3-4 days, and later the cells were passaged at the ratio of 1:3 to 1:6 every 3 days. For single-animal experiments, the cells were passaged every 3-4 days in the same medium sequentially from 96- to 48- to 24- to 12-well plates, and finally to 12.5 cm^2^ T-flasks. For amplifying cells for large-scale experiments, cM3/Extra FBS medium was used (cM3 plus 7.5% extra FBS, Invitrogen). These cell lines can be frozen in culture medium plus 10% DMSO in liquid N_2_, thawed and cultured again. Fly extract was prepared as previously reported (Currie et al., 1988), except that we homogenized 1 g of female flies in 10 ml of basic M3 serum-free medium.

### Cloning of *Ras^V12^/scrib^−/−^* cells

The cells were trypsinized and large clumps broken by repeated pipetting followed by filtering through a 25-μm mesh (Falcon). The cells were allowed to sit for 10 min to allow any clumps to settle and the supernatant containing mostly single cells was resuspended in 1× M3 containing 1% FCS. Single cells were collected by sterile FACS at the Yale FACS facility. The cells were then resuspended in cM3 medium and plated into 96-well plates to obtain a single cell/well. Of the 400 cells that were plated, 48 were successfully cloned. To select clones with a more spread-out monolayer morphology, 28 were examined and four were chosen.

### Karyotyping

Karyotype analysis followed the protocol of Cherbas and Cherbas (2007), except that we increased the vinblastine sulfate to 2 µg/ml and the cells were treated with this drug for 3-4 h.

### Transplantation assay

The cells were trypsinized, washed two times with sterile 1× PBS, and resuspended in 1× PBS with a concentration of approximately 5×10^7^ cells/ml. Transplantation experiments were performed as previously described (Pagliarini and Xu, 2003).

### RNAi treatment and screen

RNAi treatment conditions were as previously described (Mihaylov et al., 2002; Baeg et al., 2005). For the RNAi screen, *Ras^V12^*/*scrib^−/−^* cells (clone 1) were amplified in cM3/Extra FCS medium. The screen was performed in 96-well plates using the *Drosophila* dsRNA library (Ambion, Inc.). Cells were trypsinized and resuspended in 1× M3 medium with no additives and an equal number of cells were added into each well containing dsRNA. The plates were gently rocked for 1 h at room temperature. A total of 225 μl of cM3/Extra FBS was then added per well. The plates were incubated at 25°C for 3 days. On day 3, a pipet tip was used to generate a scratch wound in the lawn of cells. The plates were incubated at 25°C for 22 h and the migration of cells was monitored and imaged using a Zeiss microscope. The RNAi screen was scored based on the extent of coverage of the scratch area, and only genes that showed greater than 90% inhibition of migration were scored as positive hits. The screen results were segregated according to protein function.

### *In vitro* Transwell invasion assay

The assay was carried out using BioCoat Matrigel Invasion Chambers (BD Scientific). In total, 5×10^5^ cells were resuspended in serum-free M3 medium and grown in the Matrigel chamber. The Transwells contained cM3/FE/Ins medium as a chemoattractant. At 40 h post-incubation, the cells were stained using the HEMA 3 stain kit (Fisher Scientific).

### *In vitro* migration assay

For the VNC migration assay, tumor cells were plated at near confluency in 35 mm dishes in 1× M3 supplemented with 20% FCS and allowed to adhere overnight. Larval tissues were dissected from L3 larvae and stored in 1× M3 complete medium on ice until use. A scratch was made in the cell monolayer and the medium aspirated from the plate. Larval tissues were then placed in the scratch area, held in place with a coverslip and fresh medium added to the cells. Control and test organs were placed in the same dish to minimize variability. Each organ (control and test) was placed in duplicate. The plate was labeled to indicate which half had the controls and test discs. A grid was drawn at the bottom of the plate to serve as a guide for wounding the monolayer as well as to direct the placement of the discs/VNC. *Drosophila* imaginal discs and organs can be maintained *in vitro* overnight and still maintain their characteristics, as has been previously reported (Schubiger and Truman, 2000); hence, the migration of cells was monitored after 24 h and imaged using a Zeiss microscope (5×, 10× and 20× objectives). *Toll-6* and *lacZ* dsRNAs were synthesized using the MEGAscript RNAi kit (Ambion, Inc.) and 25 µg of RNAi used in each 35 mm tissue culture dish. Cells were treated with RNAi in 1× M3 medium for 1 h and then supplemented with 20% FCS. After 48 h, a scratch was created, and larval tissues placed in the wound area. Migration of cells was assessed after 24 h, as described above. Cells that migrated beyond 200 µm from the edge of the scratch were counted and plotted for quantification. The experiments were repeated at least three times. The 10× or 20× images were used to count cells that traversed beyond the 200 μm using Adobe Photoshop. Duplicate values from each experiment were averaged and the final three values used for statistical analysis with GraphPad.

### Plasmid construction

*Toll-6* was cloned into the *Eco*R1/*Xba* site of the pUAST vector while the activated form of *Toll-6* (*Toll-6*^ACT^) was cloned into the *Eco*RV site of pUAST (*Toll-6*^ACT^ was a kind gift from Dr J. Imler, Université de Strasbourg, CNRS, Strasbourg, France). Toll-6^ACT^ is a recombinant chimera in which the constitutively active extracellular domain of Toll is fused to the transmembrane and intracytoplasmic domains of Toll-6 (Tauszig et al., 2000). *Toll-6* cDNA was obtained from the *Drosophila* Genomics Resource Center (DGRC). The restriction sites were introduced into the fragments by PCR amplification, sequenced and inserted into a pUAST vector. RNA from different larval organs was isolated using the Trizol reagent (Invitrogen) and RT-PCR was performed using routine protocols.

### Real-time and quantitative real-time PCR

The reverse transcription of total RNA (1 µg) of *Drosophila* larval tissues into cDNA was performed using the Bio-Rad iScript cDNA Synthesis Kit following the standard protocol of the manufacturer (Bio-Rad). RT-PCR for Spz-family ligands was performed using primers (Table 1) and conditions previously described (Parker et al., 2001). *RP49* (reference gene) PCR was performed as previously described (Mishra-Gorur et al., 2002). (Larval tissues were dissected with extreme care to eliminate contamination from attached nerves, tracheal fibers or other tissues.)

**Table 1.**
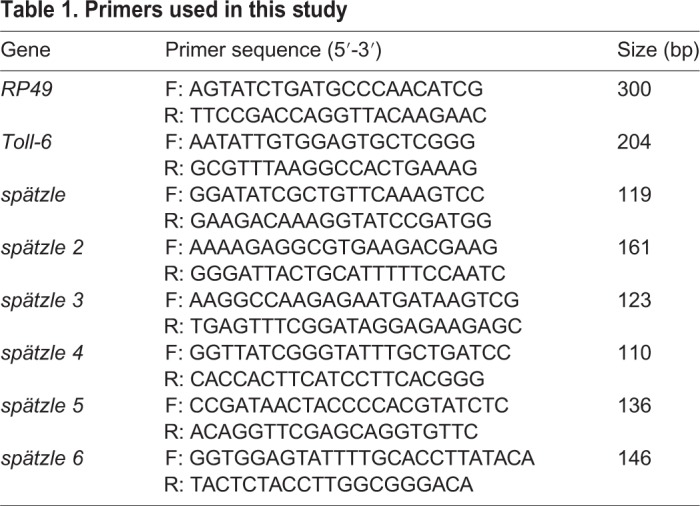
**Primers used in this study**

Quantitative real-time PCR (qPCR) primers were designed with an amplicon size range of 110-160 bp with CG clamp at the 3′ end using Primer3 software (Untergasser et al., 2012; Koressaar and Remm, 2007) and tested for specificity by blasting the sequences against the *Drosophila* genome on Ensembl BLAST. *Drosophila* *RP49* was used as a reference gene. The qPCR was performed in a total of 15 µl, containing 2 µl of cDNA, 7.5 µl of Roche FastStart Universal SYBR Green master mix, and 300 nM of the forward and reverse primers. The reaction was carried out on a Bio-Rad CFX-384 real-time PCR system under the following conditions: activation of FastStart Taq DNA polymerase at 95°C for 10 min followed by 40 cycles of 15 s denaturation at 95°C and 1 min annealing at 60°C. After this run, melting-curve analysis was performed to identify primer dimers. Genomic DNA contamination and cross-contamination were checked by including a no reverse transcriptase (no-RT) control for each sample and a no-template control for every different gene analyzed during the preparation of samples. Each sample is run in triplicates for each gene to be assayed (Schmittgen and Livak, 2008).

### Antibodies, histology and imaging

A rabbit polyclonal antibody was generated against the intracellular domain of Toll-6. Two C-terminal peptides in Toll 6 (GC-SLNDDEDEDHDQQKNLWA and GC-TLEHQHHHNHQANRRSQH) were used for injecting into rabbits (Cocalico, Inc.). The antibody was affinity purified and used at a 1:50 dilution for antibody staining. The specificity of the anti-Toll-6 antibody was confirmed by immunostaining of either S2 cells or wing imaginal discs expressing Toll-6. Larval tissues were stained with standard immunohistochemical procedures using rabbit anti-MMP-1 antibody (1:40; DSHB) or rabbit anti-phospho-JNK polyclonal antibody (1:50; Calbiochem), mouse anti-beta-gal antibody (1:200; Sigma), secondary Alexa-Fluor-555- or Alexa-Fluor-488-conjugated antibodies (1:500 dilution for 90 min; Invitrogen). Tissues were mounted in a drop of Vectashield-DAPI (Vector Laboratories, Burlingame, CA, USA). A Zeiss LSM510 Meta Confocal Microscope (Zeiss, Jena, Germany) was used for analysis. In all experiments, identical confocal settings were used for imaging when samples were being compared.

### Western blot

Tumors were dissected and homogenized in Tris lysis buffer (50 mM Tris, pH 7.4; 150 mM NaCl; 1 mM EDTA; 1% NP-40). The samples were run on 10% SDS-PAGE gels (Bio-Rad) and western blots were performed according to standard methods. In brief, lysates were separated on 10% SDS-PAGE gels and transferred to nitrocellulose membranes. After blocking with 5% milk, the membranes were incubated first with polyclonal antibodies against phosphor-JNK (Calbiochem) or tubulin (DSHB), and then with a secondary HRP-conjugated anti-rabbit IgG (Jackson ImmunoResearch). Signals were detected with an ECL-plus kit (PerkinElmer).

## Supplementary Material

Supplementary information
