# Supplementary material for: Spz/Toll-6 signal guides organotropic metastasis in Drosophila
Source: Dis Model Mech. 2019 Oct 7;12(10):dmm039727. doi: 10.1242/dmm.039727 (PMC6826028; doi:10.1242/dmm.039727)
Supplement: Supplementary information [file dmm-12-039727-s1.pdf]

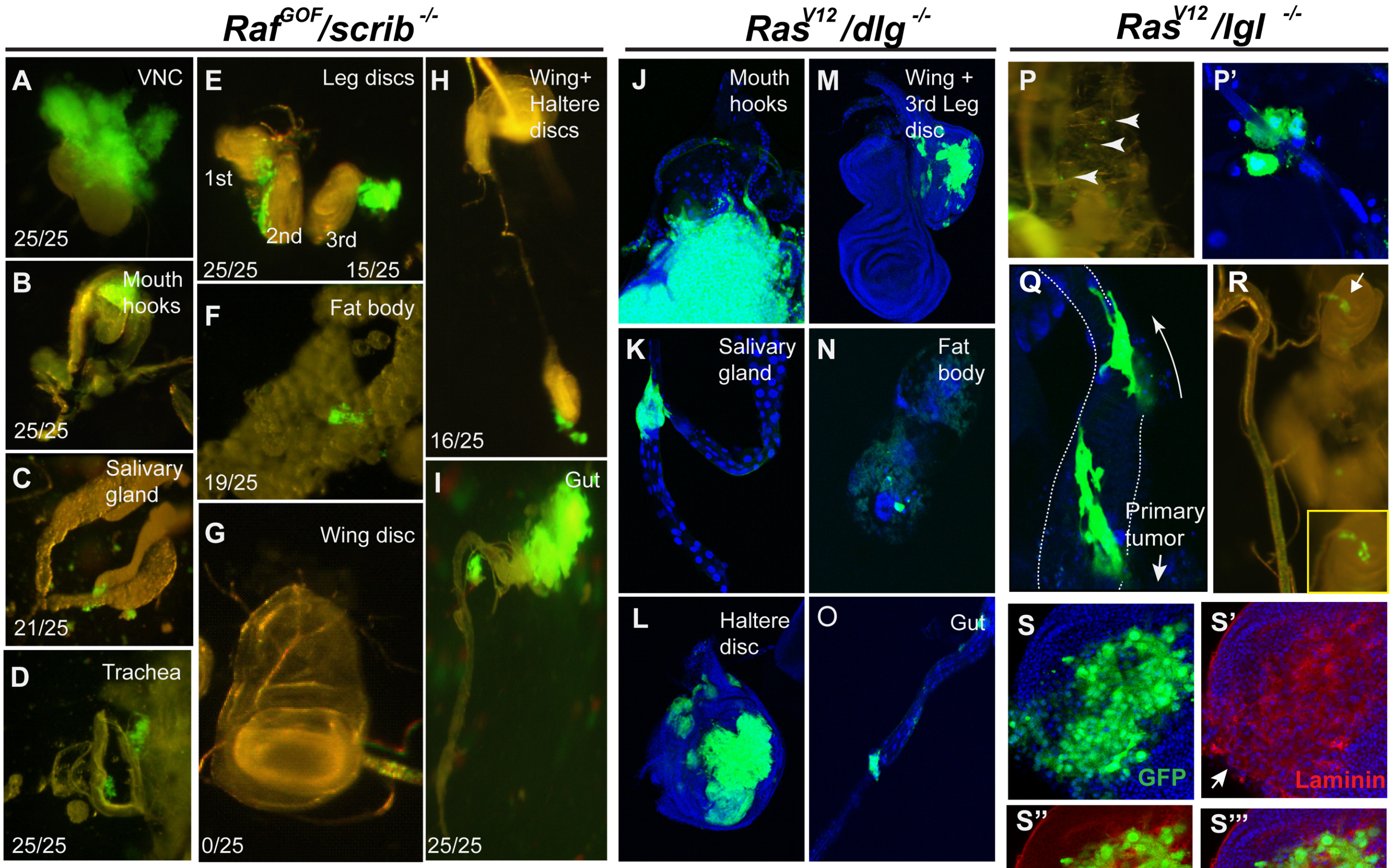

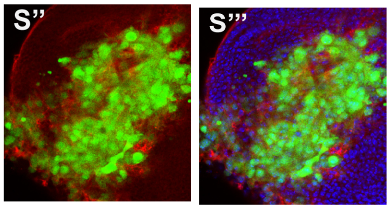

Organotropic behavior of ***$Raf^{GOF}/scrib^{-/-}$*** **and** ***$Ras^{V12}/Dlg^{-/-}$*** **tumors:** GFP labeled *$Raf^{GOF}/scrib^{-/-}$* and *$Ras^{V12}/Dlg^{-/-}$* clones were generated in eye discs, the ensuing clones develop into tumors and migrate onto the VNC (A), mouth hooks (B,J), salivary glands (C,K), trachea (D), leg discs (E,M), haltere disc (H,L), fat body (F,N), gut (I, O). Tumor cells do not migrate onto the wing disc (G,M). Numbers indicate number of larvae displaying invasion of the organ out of total number of larvae dissected. Confocal images of invasion of *$Ras^{V12}/Dlg^{-/-}$* metastasis (J-O). Light (P, R) and confocal (P', Q) microscope images showing tumor cells are primarily associated with the tracheal network (arrow head), trachea is outlined for clear visualization, Primary tumor is out of the field and arrow indicatesdirection of migration. Cells move off the trachea onto the surface of a leg disc (R). Confocal image of leg disc with laminin staining for basement membrane shows disruption (arrow) indicating invasion (S-S"). Numbers indicate number of larvae displaying metastasis to the organ examined/ total number of larvae dissected

Supplementary Information: Figure S1

Disease Models & Mechanisms • Supplementary information

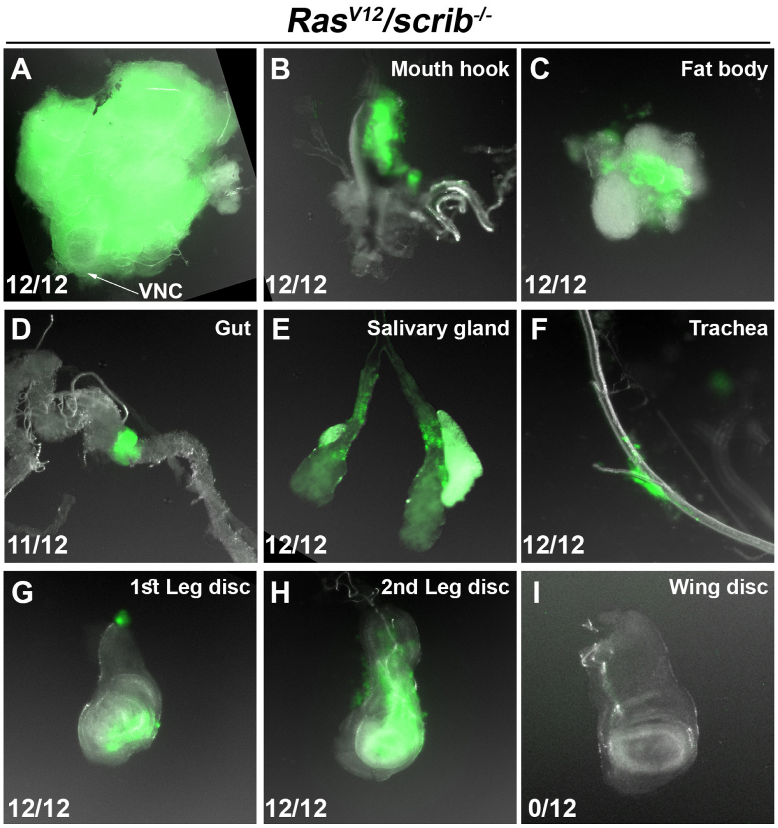

Supplementary Information:Figure S2

Oraganotropic behavior of RasV12/scrib-/- tumors: GFP labeled RasV12/scrib-/- clones were generated in eye discs, the ensuing clones develop into tumors and migrate onto the VNC (A), mouth hooks (B), fat body (C), Gut (D), salivary gland (E), trachea (F) and leg discs (G,H). Tumor cells do not migrate to the wing disc. Numbers in each panel indicate number of larvae displaying metastasis to the organ out of the total larvae dissected.

Disease Models & Mechanisms • Supplementary information

Supplementary Information: Figure S3

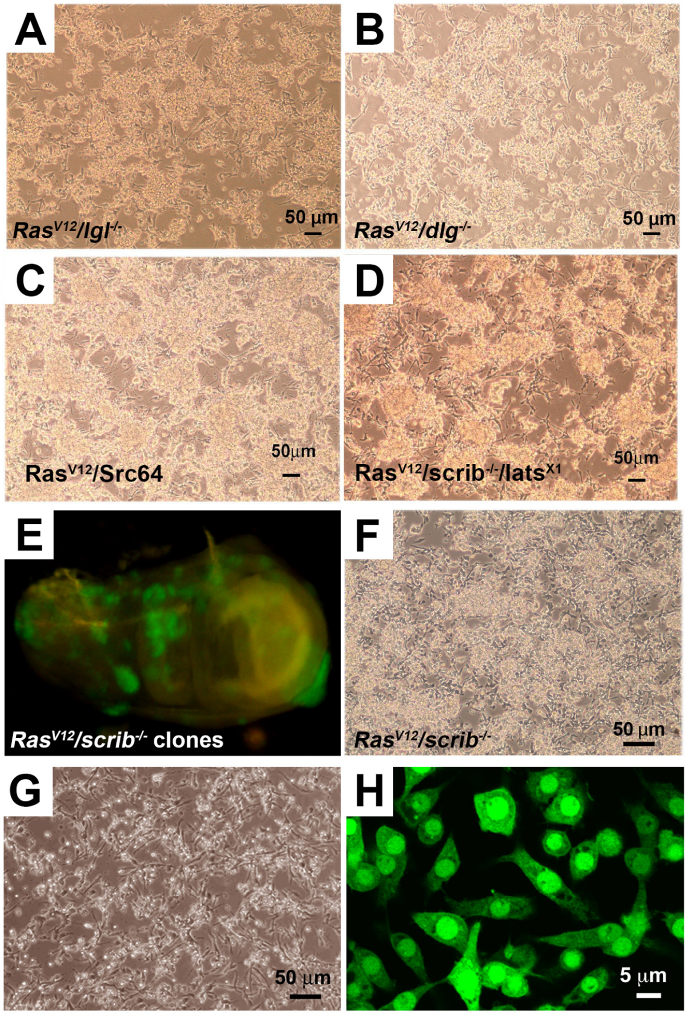

Fly tumor cell lines characterization.
(A) Ras^{V12}/lgl$^{-/-}$ tumor cell lines. (B) Ras^{V12} /dlg$^{-/-}$ tumor cell lines. (C) Ras^{V12}/Src64 tumor cell lines. (D) Ras^{V12}/scrib$^{-/-}$/lats^{X1} tumor cell lines. (E) Ras^{V12}/scrib$^{-/-}$ mutant clones (GFP-positive) are induced by heat shock in the wing disc. (F) Ras^{V12}/scrib$^{-/-}$ cells derived from the wing disc tumor clones. (G) Cloned Ras^{V12}/scrib$^{-/-}$ cells in cM3 media supplemented with fly extract and insulin (cM3/FE/Ins). (H) High magnification confocal image of cells in (G).

Disease Models & Mechanisms • Supplementary information

Supplementary Information: Figure S4

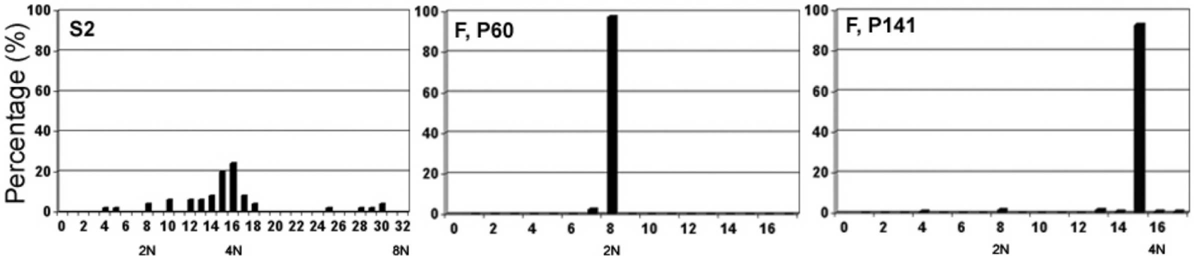

Chromosome number distribution of S2 cells (left), $Ras^{V12}/scrib^{-/-}$ female cells at P60 (middle) and at P141 (right).

Disease Models & Mechanisms • Supplementary information

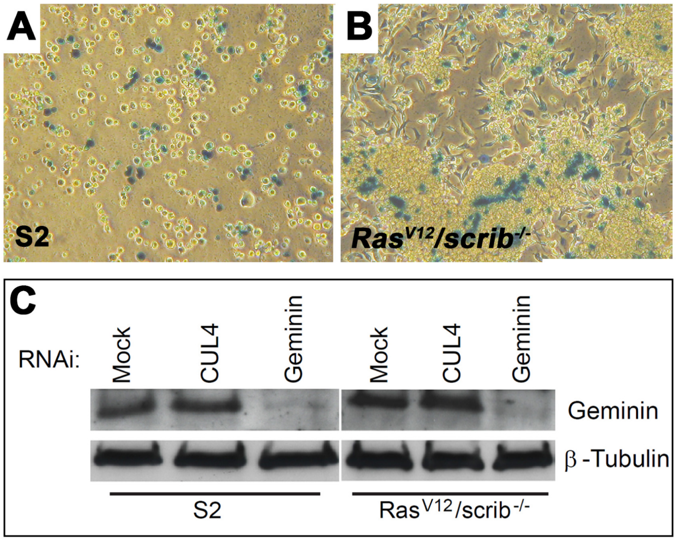

Supplementary Information: Figure S5

Ras^{V12}/*scrib*$^{-/-}$ tumor cells are amenable to transfection and RNAi treatment. (A-B) S2 cells (A) and (B) *Ras*^{V12}/*scrib*$^{-/-}$ cells display similar transfection efficiency. Cells are transfected with a lacZ reporter and stained for β-Galactosidase. (C) Geminin protein were strongly downregulated by geminin RNAi treatment in both S2 cells and *Ras*^{V12}/*scrib*$^{-/-}$ cells, but not by CUL4 RNAi. β-Tubulin was used as a loading control.

Supplementary Information: Figure S6

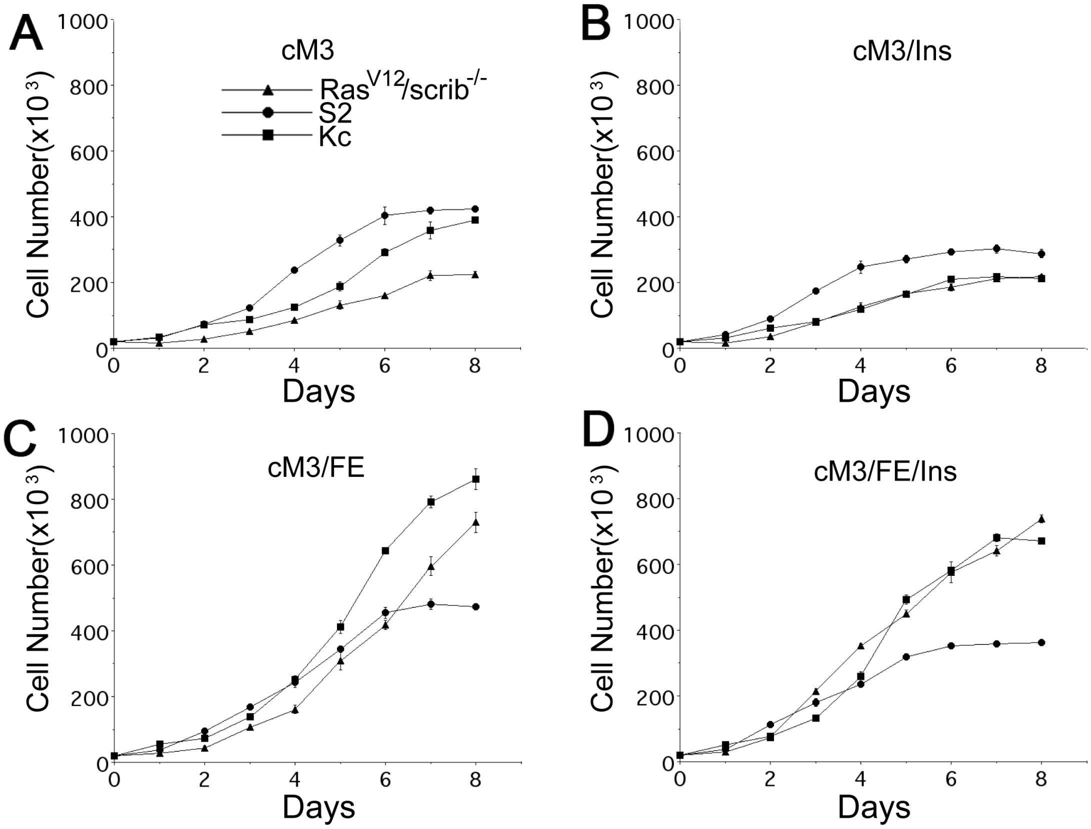

Growth properties of ***Ras^{V12}/scrib$^{-/-}$*** cells.
Ras^{V12}/scrib$^{-/-}$ (▲), S2 (●) and Kc (■) cells. (A) Cells grown in cM3 medium, (B) Cells grown in cM3 medium supplemented with insulin (cM3/Ins), (C) Cells grown in cM3 medium supplemented with fly extract (cM3/FE), (D) Cells grown in cM3 medium supplemented with fly extract and insulin (cM3/FE/Ins). experiment was done 3 times and averages used to caculate STD error.

Supplemental Information: Figure S7

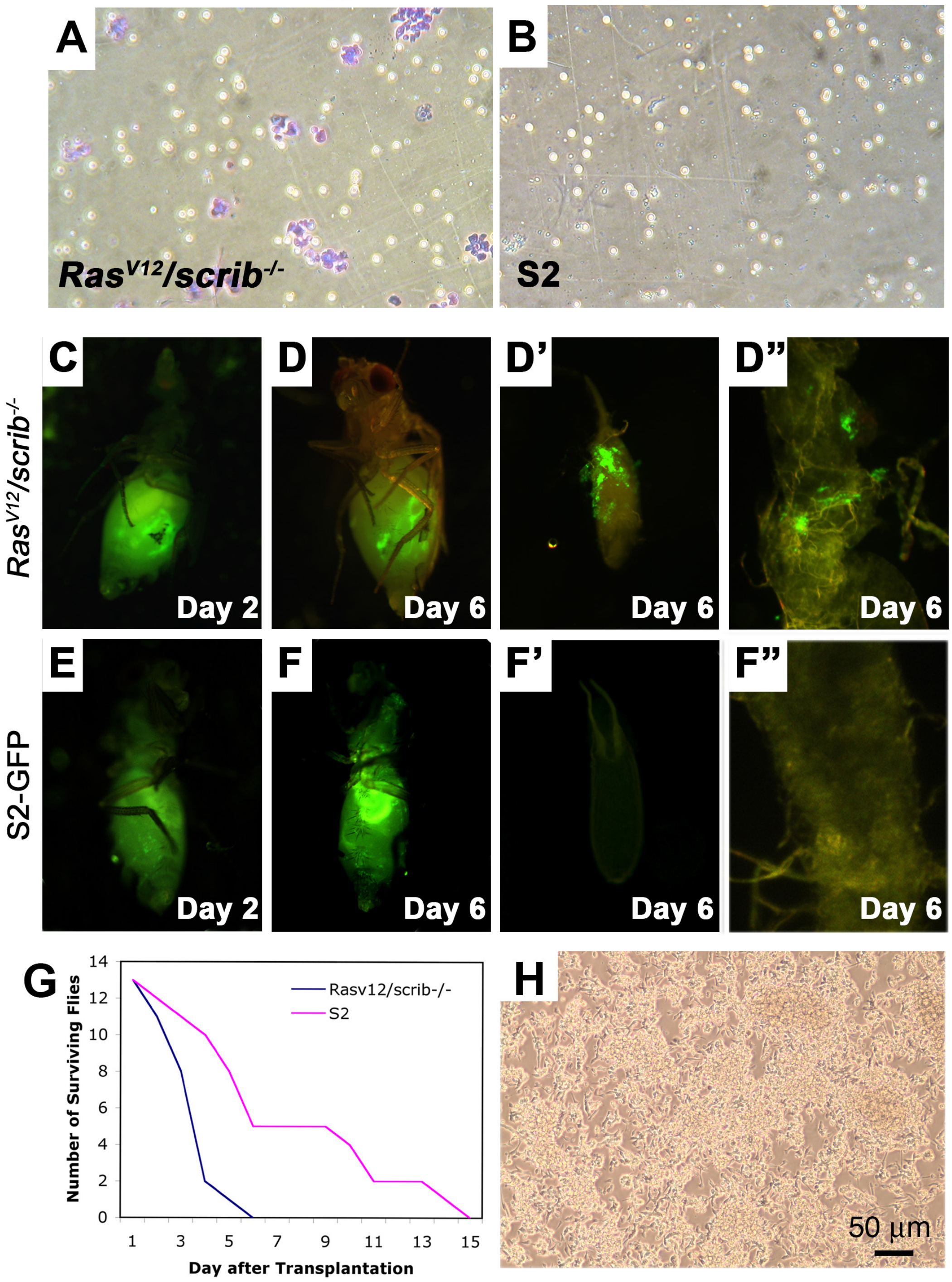

Ras^{V12}/scrib$^{-/-}$ tumor cells display invasive properties in vitro and in vivo.
(A-B) Ras^{V12}/scrib$^{-/-}$ tumor cells (A), but not S2 cells (B), invade Matrigel after 40 hrs in the in vitro transwell invasion assay. (C-F”) Transplantation of cells into adult female flies. Ras^{V12}/scrib$^{-/-}$ cells (GFP-positive, C-D”), but not S2 cells (GFP-positive, E-F”) invade the gut and ovary upon transplantation. Adult female flies at day 2 (C, E) and Day 6 (D, F) after transplantation; and the dissected out ovarian follicles (D’, F’) and intestine (D”, F”). (G) Survival curve of transplanted flies with corresponding cell lines. (H) The Ras^{V12}/scrib$^{-/-}$ cells from transplanted flies can be re-isolated and cultured again in vitro.

Disease Models & Mechanisms • Supplementary information

Supplemental Information: Figure S8

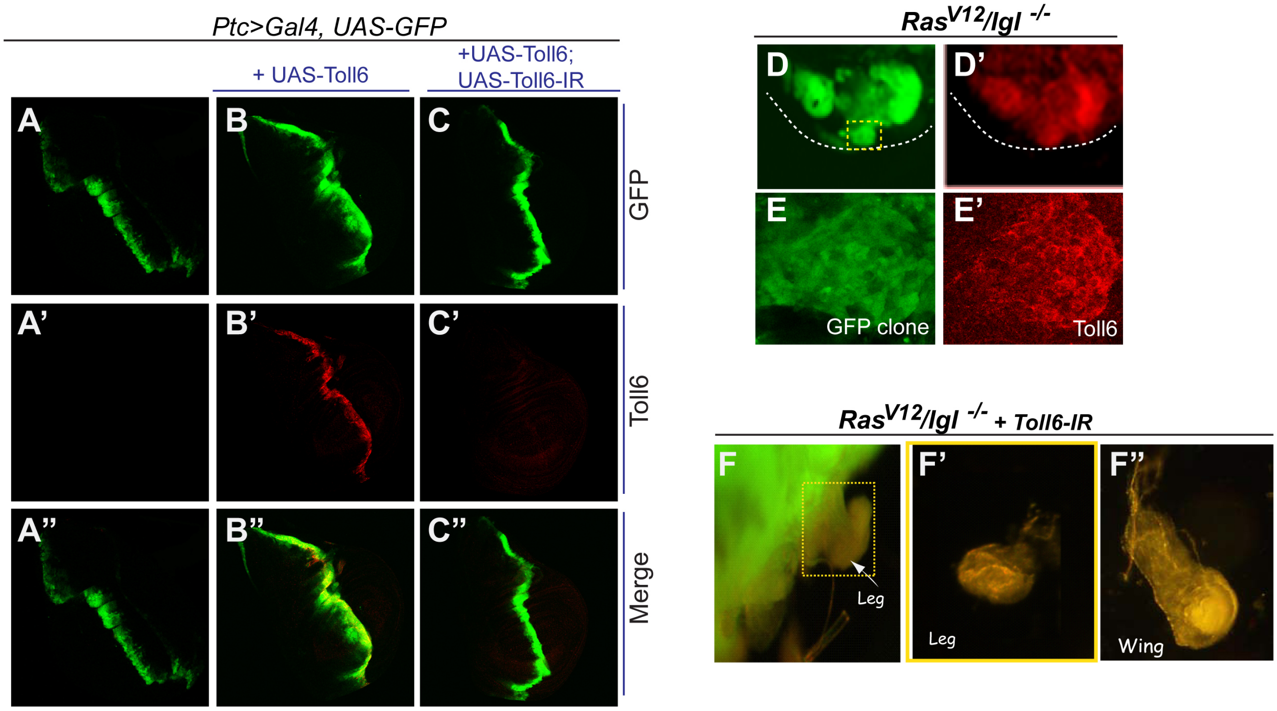

A-C": Specificity of Toll6 antibody: Toll6 was expressed in the wing disc under the Ptc>Gal4 driver and discs stained with rabbit Toll6 antibody. Co-expression of Toll6-IR abolishes staining of the protein. All images captured using identical Confocal settings. D-D': RasV12/lgl-/- clones show Toll6 expression. E-E": Higher magnification image of GFP clone, All cells in the clones do not express equal amounts of Toll6. F-F": RasV12/lgl-/-, Toll6IR tumors contact other organs (F) but do not invade them: e.g. leg disc: dissected away from a Toll-6IR tumor (F') does not show any tumor cells and looks free of tumor cells like the wing disc dissecetd from the same larva (F").

Disease Models & Mechanisms • Supplementary information

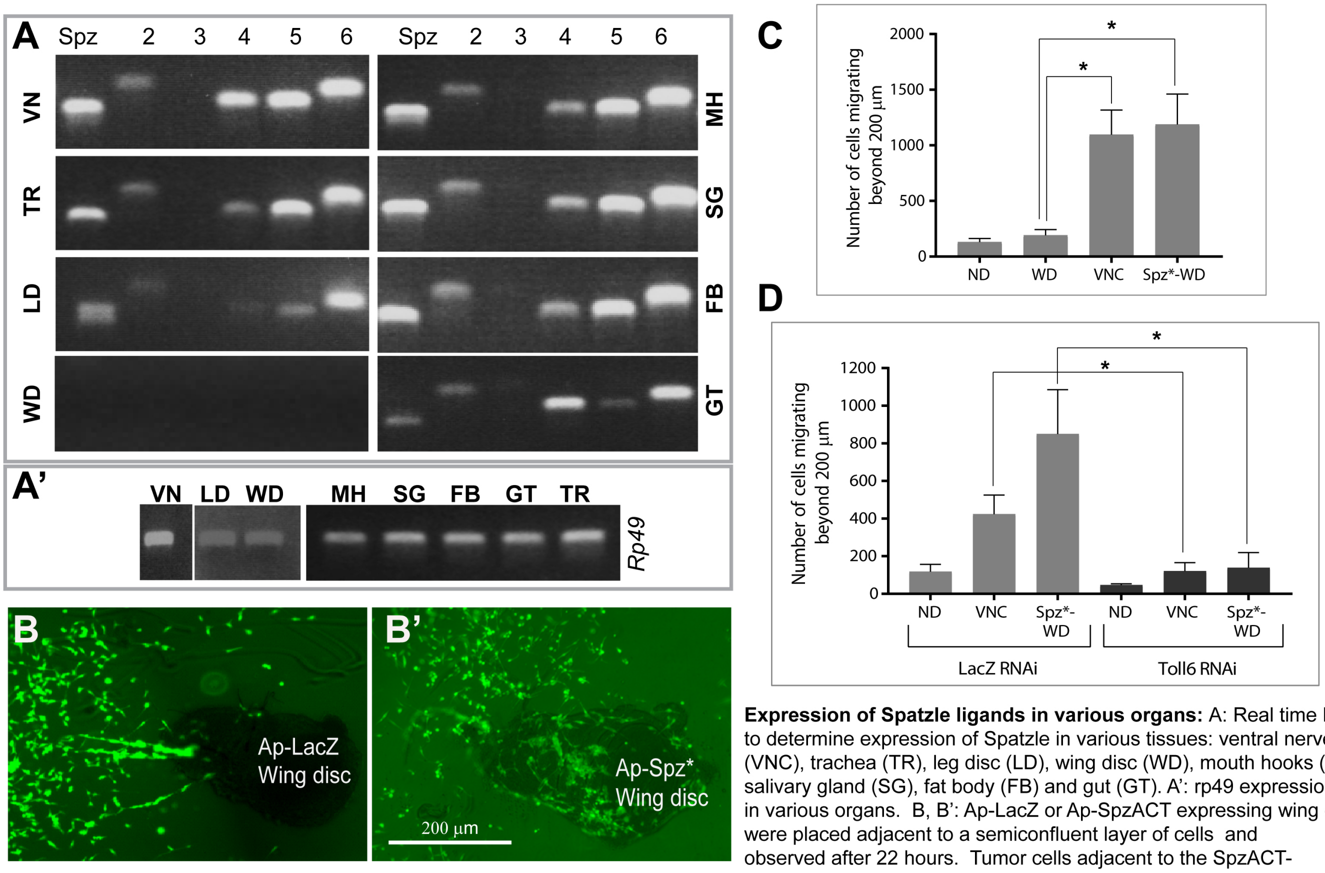

Expression of Spatzle ligands in various organs: A: Real time PCR to determine expression of Spatzle in various tissues: ventral nerve cord (VNC), trachea (TR), leg disc (LD), wing disc (WD), mouth hooks (MH), salivary gland (SG), fat body (FB) and gut (GT). A': rp49 expression in various organs. B, B': Ap-LacZ or Ap-SpzACT expressing wing discs were placed adjacent to a semiconfluent layer of cells and observed after 22 hours. Tumor cells adjacent to the SpzACT-expressing wing disc but not LacZ-expressing wing disc migrated onto the disc and accumulated on the tissue. For the LacZ expressing disc, the cells stayed on the attached tissue (nerves, trachea) and did not migrate onto the disc surface for upto 96 hrs. Quantification of cell migration in vitro: C,D: Quantification of tumor cells migrating towards organs in the in vitro scratch assay. cells that migrated beyond 200 microns towards organs from the edge of the scartch were counted from three independent experiments and graphed. C: Cells quantified in the presence of no disc, wing disc, VNC and SpzACT-expressing wing disc. Unpaired t-test with Welch's correction: *: *p<0.02 (*Two-tailed)*,* N= 3 independent repeats (in duplicates). D: Cells were treated with either LacZ or Toll6 RNAi prior to wounding. Migration of cells after RNAi treatment was assessed in response to no disc, VNC and SpzACT-expressing wing disc.

Supplemental Information: Figure S9

Disease Models & Mechanisms • Supplementary information

Supplemental Information: Figure S10

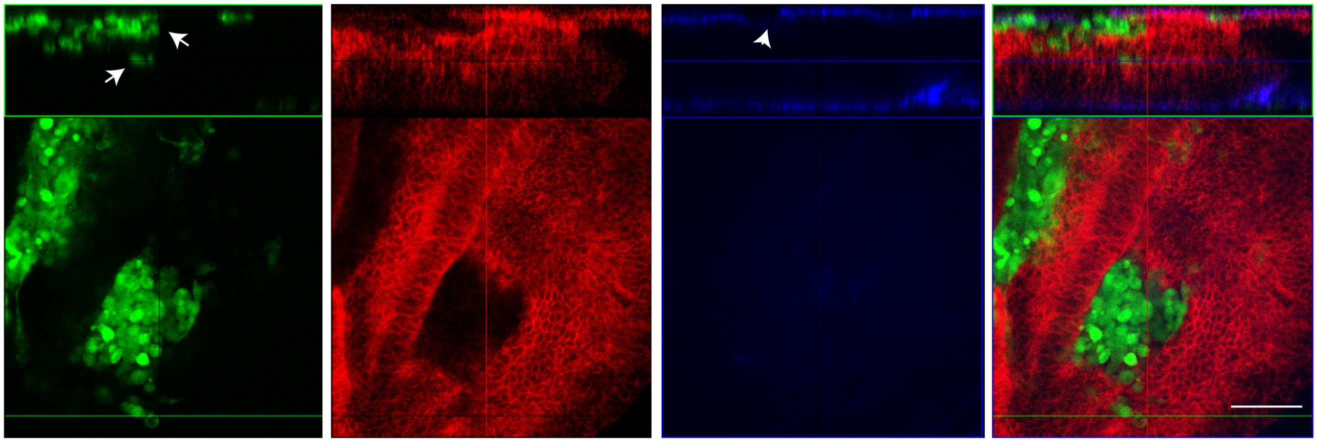

Invasion of SpzACT-expressing wing disc by tumor cells in vitro: In the in vitro scratch assay, cells migrate towards wing discs expressing activated spatzle under the Nubbin driver (*Nub-SpzACT, UAS-RFP*). After 24 hrs, the disc was fixed and stained for laminin. Invasion of tumor cells into the disc was assessed by Confocal microscopy. Degradation of the basement membrane is clearly visible by the break in laminin staining (arrowhead) and GFP-positive tumor cells can be seen invading into the disc. Scale bar: 100 µm

Disease Models & Mechanisms • Supplementary information

Supplemental Information: Figure S11

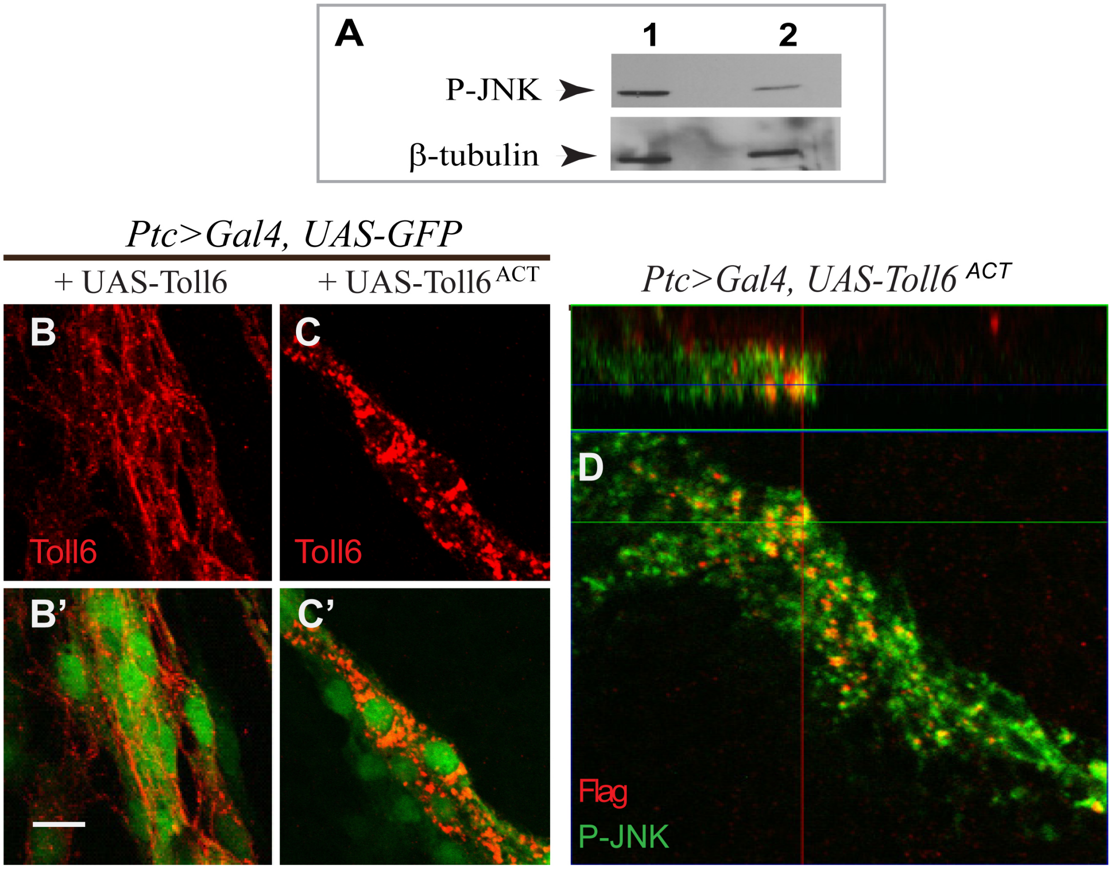

Toll-6 activates JNK. A: Western analysis of tumors shows an approx. 40% reduction in levels of phosphorylated JNK (H). Lane 1: *RasV12/lgl*$^{-/-}$ tumors, Lane 2: *RasV12/lgl*$^{-/-}$, *Toll6-IR* tumors. Bottom panel shows staining for β-tubulin as a loading control. Ectopic expression of Toll-6 or Toll6^{ACT}-Flag under the Patched driver (*ptc>Gal4*) in wing discs shows a differential localization pattern. Full length Toll6 localizes to the cell surface (B, B') while activated Toll-6^{ACT} displays a particulate pattern (C, C'). D: Co-immunostaining for P-JNK and Flag shows co-localization of P-JNK and Toll-6^{ACT}.

Supplemental Information: Figure S12

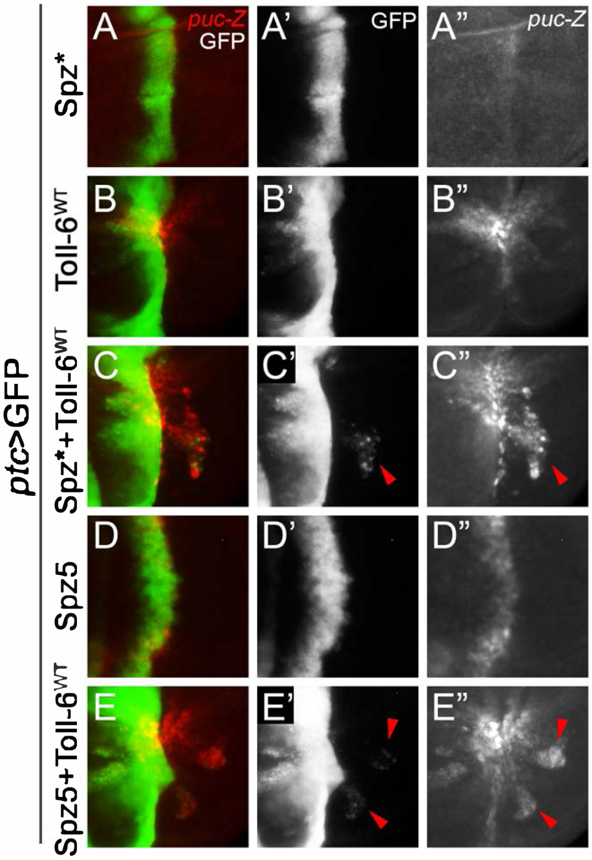

Spz and Toll-6 synergistically induce collective cell migration. Fluorescence micrographs of Drosophila wing pouch regions are shown. Expression of Spz, Spz5 or Toll-6 alone under ptc promoter cannot promote cell migration (A', D', B'), whereas co-expression of Spz and Toll-6 or Spz5 and Toll-6 significantly promotes collective cell migration (C', E'), indicated by red arrow heads. Note that expression of Spz5 (D"), but not Spz (A"), is sufficient to induce puc transcription, a readout of JNK activation.

Disease Models & Mechanisms • Supplementary information
